# Optimization of the antifungal metabolite production in *Streptomyces libani* isolated from northern forests soils in Iran

**DOI:** 10.18502/cmm.6.4.5333

**Published:** 2020-12

**Authors:** Maryam Azish, Masoomeh Shams Ghahfarokhi, Mehdi Razzaghi Abyaneh

**Affiliations:** 1 Department of Mycology, Faculty of Medical Sciences, Tarbiat Modares University, Tehran, Iran; 2 Department of Mycology, Pasteur Institute of Iran, Tehran, Iran

**Keywords:** Antifungal metabolite, Aspergillus spp., Soil bacteria, *Streptomyces libani*

## Abstract

**Background and Purpose:**

Soil bacteria have extreme population diversity among natural sources and are able to produce a wide array of antifungal metabolites. This study aimed to isolate and identify the bioactive metabolite-producing bacteria from forest soils and evaluate their antimicrobial potent against some pathogenic organisms.

**Materials and Methods:**

In this study, soil samples were screened for antifungal activity against *Aspergillus fumigatus* on glucose-yeast extract (GY) agar using a visual agar plate assay method. All growing bacteria were examined for antifungal activity, and antagonistic bacteria were identified based on 16S ribosomal RNA sequence analysis. For optimization of the production of antifungal bioactive metabolites, inhibitory bacteria were cultured on different culture conditions, including media, pH, temperature, and incubation time.

**Results:**

In total, 110 bacterial strains were isolated from the forest soils and four species with high antifungal activity were identified as *Streptomyces libani, Streptomyces angustmyceticus, Bacillus subtilis,* and *Sphingopyxis* spp. on the basis of 16s ribosomal RNA sequencing. Dichloromethane extract of the starch casein broth culture filtrate of the S. libani (incubated at 30° C for five days) showed strong antifungal activity against *A. fumigatus, Aspergillus niger,* and *Aspergillus flavus*.

**Conclusion:**

Based on the results, forest soils contain organisms with antifungal activity and could be considered as a good source for novel antifungal metabolites as effective and safe therapeutics.

## Introduction

spergillosis is an infection caused by Aspergillus species. Most commonly, it occurs in the form of chronic pulmonary aspergillosis (CPA), aspergilloma, allergic bronchop-ulmonary aspergillosis (ABPA), and invasive aspergillosis [ 1 ]. Invasive form of the disease only occurs in severely immunocompromised patients, such as organ transplant recipients, hematopoietic stem cell transport, and patients taking chemotherapy or taking corticosteroids [ 2 ]. Aspergillus fumigatus is the most prevalent etiologic species that is mainly responsible for increasing the incidence of invasive aspergillosis in immunocompromised patients [ 3 ]. Invasive aspergillosis has been reported in 2-26% and 1-15% of hematopoietic stem cell and organ transplant recipients, respectively [ 4 ]. Synthetic antifungal medications and fungicides, particularly azole group medications, have been used to treat aspergillosis [ 1 , 5 ]. Despite the effectiveness of azoles, polyenes, and echinocandins as therapeutics for clinical forms of aspergillosis, there are still some limitations, including life-threatening side effects, an increase of the antifungal resistant species, and undesirable effects on non-target beneficial microorganisms that are shared in the ecosystem [ 1 , 5 , 6 ]. Amphotericin B; newly developed azoles, such as voriconazole and posaconazole; and the echinocandins are active against Aspergillus spp. However, the resistance of Aspergillus isolates to amphotericin B, itraconazole, voriconazole, and caspofungin have also been reported in some patients [ 1 , 5 , 6 ]. Management of invasive aspergillosis continues to be challenging, and the mortality rate remains unacceptably high; therefore, there is a need for the development of new, safe, and more effective antifungal antibiotics that will reduce the damage to human health and the ecosystem around them. 

In recent years, natural products have widely been considered for the development of new antimicrobial agents with the potential for the treatment of fungal infections [ 7 ]. According to the Antimicrobial Peptide Database, 959 antifungal peptides have been isolated and chemically synthesized from various sources, such as soil bacteria [ 8 ]. Soil bacteria have received major consideration due to their extreme population diversity and production of a variety of antifungal compounds. They mainly belong to the genera *Streptomyces*, Bacillus, and Pseudomonas [ 9 - 13 ]. According to different studies, the genus *Streptomyces* produces approximately 75-80% of the total antibiotics produced by microbes, such as nystatin, amphotericin B, natamycin, bafilomycin A1, concanamycin, and 3-phenylpropionic acid [ 14 - 16 ]. This study aimed to screen and identify the antifungal metabolites-producing bacteria isolated from Northern forest soils in Iran and evaluate their antimicrobial potent against some pathogenic organisms. 

## Materials and Methods

**Forest soil sampling**

In total, 45 soil samples were collected from five various locations from the northern forests in Babol (Latitude: 36° 32' 59.99" N Longitude: 52° 40' 59.99" E) and Sari (Latitude: 36° 33' 47.95" N Longitude: 53° 3' 36.32" E), Iran. These soil samples (200 g) were collected from the depth of 15-20 cm ground surface. All the samples were packed in sterile zip bags and kept at 4ºC [ 17 ]. 

**Fungal and bacterial strains**

The fungal strains, including *A. fumigatus* Af239, *A. flavus* PFCC 127, Aspergillus niger PFCC 92-844, *Candida* albicans ATCC 10231, *Candida* glabrata ATCC 90030, and Cryptococcus neoformans PFCC 93-589, were obtained from pathogenic fungi culture collection of the Department of Mycology, Pasteur Institute of Iran (http://fa1.pasteur.ac.ir/pages.aspx? id=1152) in Tehran, Iran. The bacterial strains, including *Escherichia coli* (ATCC 25922) and *Staphylococcus aureus* (ATCC 6538), were obtained from the culture collection of the Department of Microbiology, Tarbiat Modares University in Tehran, Iran. *The fungal isolates were* cultured on Sabouraud dextrose agar (SDA) (manufactured in Merck, Germany) at 28ºC for 4 days and *bacterial isolates were* cultured on nutrient agar (manufactured in Merck, Germany) at 30ºC for 24 h. The spore suspension was prepared by sterile phosphate-buffered saline (PBS) in the final concentration of 2×10^5^ spores/ml for filamentous fungi. Yeast cells of *Candida* and Cryptococcus species were adjusted to 0.5-2.5×10^3^ cells/ml of PBS, while bacteria suspensions were prepared in 0.5 McFarland concentration [ 17 ]. 

**In search of antifungal metabolite-producing organisms in soil samples**

An amount of 0.1 g of each soil sample was dissolved in 1ml of sterile PBS, vortexed for 30 s, and centrifuged at 2500 rpm for 10 min. The supernatant was tested for antifungal activity against *A. fumigatus* by visual agar plate assay according to Ranjbariyan et al. [ 13 ]. Moreover, 3 μl of spore suspension of A. fumigatus (2×10^5^ spores/ml) was inoculated on the center of glycerol casein agar medium (GCA) plate, including glycerol (10 g), casein (0.3 g), K_2_HPO_4_ (2 g), KNO_3_ (2 g), NaCl (2 g), MgSO_4_ (0.05 g), CaCO_3_ (0.02 g), FeSO_4_ (0.01 g), agar (18 g), pH=7). Besides, 10 μl of the supernatant of each soil suspension was placed peripheral around the plate and the plates were incubated for 96 h at 28˚ C [ 18 ]. 

**Isolation of bacteria with antifungal activity from bioactive soil samples**

Soil samples with antifungal activity in initial screening were cultured on GCA and incubated at 30º C for seven days. Inhibitory effect of bacterial isolates against *A. fumigatus* growth was tested by visual agar plate assay according to Ranjbariyan et al. [ 13 ]. The antifungal activity of each bacterium was analyzed by determining the fungal growth zone of inhibition. The bacteria with the highest activity were stored in 20% glycerol (20%, v/v) at -20° C. 

**Molecular identification of antifungal-producing bacteria (DNA extraction, PCR, and sequencing)**

The DNA extraction was performed according to Kumar et al. with slight modifications [ 19 ]. The selected antifungal-producing bacteria were inoculated in 100 ml starch casein broth for four days at 28º C, pH 7 in 500 ml flasks on a rotary shaker (120 rpm). The media were centrifuged (8000 rpm for 6 min) and biomass was harvested, washed twice with sterile distilled water, and suspended in 800 µl lysis buffer (100 mM Tris-Hcl, pH=7, 20 mM EDTA, 250 mM NaCl, 2% SDS, and 1mg/ml lysozyme). Purity of genomic DNA was checked by gel electrophoresis 1% agarose. The 16S ribosomal DNA sequences were amplified using 0.5 µl from universal primers 27F (5′-AGAGTTTGATCCTGGCTCAG-3′) and 1492R (5′-CGGTTACCTTGTTACGACTT-3′), 12.5 µl master mix, 1 µl DNA, and 10.5 µl distilled water in a thermal cycler (TC-E-96G, China). The conditions were as follows: initial denaturation at 95° C for five min, followed by 35 cycles of denaturation at 94° C for 30 s, annealing at 58° C for 30 s, extension at 72° C for 70 s, and a final extension at 72°C for five min; eventually, the PCR product was kept at 4°C. The amplified products were confirmed by electrophoresis on 1% agarose gel [ 20 ]. 

The PCR products were sequenced using the forward primer which was used for amplification by ABI prism Big Dye Terminator v3.1 Cycle Sequencing Kit (manufactured in Applied Biosystems, USA). The highest sequence similarity of the 16s ribosomal RNA sequence of isolates with the reference species of bacteria was confirmed by using the NCBI BLAST tool (http://WWW.ncbi.nlm.nih.gov/ BLAST). 

**Optimization of antifungal compounds produced by inhibitory bacteria**

In order to optimize the cultural conditions and to identify the best culture broth for the production of inhibitory bioactive metabolite against the different pathogens, the bacteria with the ability to produce antifungal compounds were inoculated in 50 ml of starch casein broth (SCB) (starch: 10g, casein: 0.3g, K_2_HPO_4_: 2g, KNO_3_: 2 g, NaCl: 2 g, MgSO_4_: 0.05 g, CaCO_3_: 0.02 g, and FeSO_4_: 0.01 g) medium and incubated at 30º C for 48 h with rotary shaking at 200 rpm [ 18 ]. Afterward, 12.5 ml of pre-cultures (3×10^5^ CFU/ml) were inoculated into 250 ml several broth media, including SCB, ISP-2B (yeast extract: 4 g, malt extract: 10 g, and dextrose: 4 g), and GYB (yeast extract 5 g, glucose 20 g) at pH 5, 7, and 9 for 5, 7, and 9 days at 28º C, 30º C, and 32º C, respectively, in 1000 ml-Erlenmeyer flasks and were incubated in a rotary shaker at 200 rpm. It should be noted that all cultures were developed in triplicate sets. The cultures were centrifuged at 10000 rpm for 10-15 min, filtered through a 0.2 µm filter membrane, and kept at 4º C [ 21 ]. 

**Evaluation of the antifungal activity of optimized culture filtrate**

The antifungal activity of all culture filtrates was examined against *A. fumigatus* by the agar well diffusion method [ 22 ]. Fungal strains (2×10^5^ spores/ml) were swab cultured on the SDA plates. Wells with diameters of 6 mm were made in the center of SDA plates, filled with 100 µl of bacteria culture filtrates, and incubated at 30º C for 5 days. The SCB, ISP-2B, and GYB media without organism were used as control. Zones of fungal growth inhibition around the central well were measured at the end of the incubation period. It should be noted that all experiments were performed in triplicate sets. 

**Solvent extraction of antifungal compounds from *Streptomyces* libani**

Antifungal metabolites were extracted using several organic solvents, including ethyl acetate (EtOAc), acetone (AC), dichloromethane (DCM), ethanol (EtOH), and methanol (MeOH). For this purpose, the solvents were separately added to the culture filtrate in the ratio of 1:1 (v/v), shaken vigorously for 45 min, and concentrated using a rotary evaporator (IKA WERKE, Germany) at 45° C [ 22 , 23 ]. The residues were dissolved in 1 ml of MeOH and examined against *A. fumigatus*, *A. flavus*, *A. niger*, *C. albicans*, *C. glabrata*, *E. coli*, and *S. aureus* using the agar well diffusion method as described above while methanol was used as the only solvent control. 

**Statistical analysis**

The collected data were analyzed in GraphPad Prism software (version 6) using ANOVA at a significance level of less than 0.05. 

## Results

**Soil sampling and isolation of bacteria with antifungal activity from selected soil samples**

**Figure 1:**
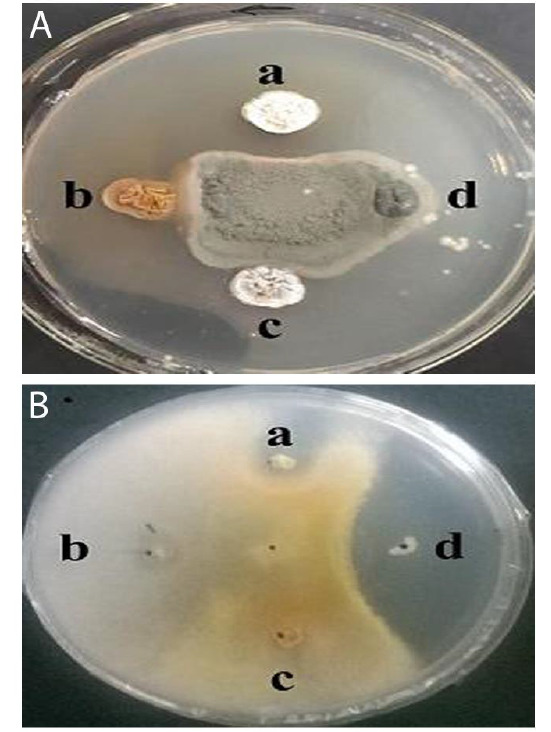
A) Soil samples screening for antagonistic activity against *Aspergillus fumigatus* on starch casein agar. a: inhibitory soils and b, c, d: non-inhibitory soils B) Isolated bacteria screening for antagonistic activity against *Aspergillus fumigatus* on CGA plate. a, b, c: non-inhibitory bacterial isolates and d: bacterial isolate with inhibitory activity regarding *Aspergillus fumigatus* growth

Out of the 45 soil samples screened for antifungal activity against *A. fumigatus*., 30 soil samples (66.66%) were found to have an inhibitory effect against *A. fumigatus*. Figure 1A shows the inhibitory (a) and non-inhibitory (b, c, and d) soils in the initial screening against *A. fumigatus*. Soils that contained inhibitor organisms were isolated on the basis of their morphological characteristics on GCA plates and gram staining. A total number of 110 bacteria isolated from 30 samples of inhibitory soils were classified into four groups, including high (4 isolates, 3.6%), moderate (40 isolates, 36.36%), low (21 isolates, 19.09%), and negative (45 isolates, 40.9%) activity, based on the visual agar plate assay (Table 1). As shown in Figure 1B, a clear zone of fungal growth inhibition is obvious for one bacterium (d), while the other three tested bacteria (a, b, and c) were not able to inhibit fungal growth. Finally, four isolates named G-92, H-57, I-65, and I-71 with strong inhibitory activity against *A. fumigatus* growth were selected for further studies. 

**Table 1:** Preliminary screening of bacteria with activity against Aspergillus fumigatus

No	Negative	Low	Moderate	High	No	Negative	Low	Moderate	High	No	Negative	Low	Moderate	High
A-1			++		A-62	-				C-107			++	
A-2	-				A-63		+			C-108	-			
A-3			++		A-64	-				C-109			++	
A-4			++		I-65				+++	C-110	-			
A-5	-				I-66	-				C-111	-			
B-6	-				I-67		+			A-122	-			
B-7			++		I-68	-				A-123		+		
B-8	-				I-69		+			A-124			++	
B-9			++		I-70	-				A-125		+		
B-10	-				I-71				+++	A-126	-			
B-11	-				H-72		+			A-127		+		
C-12		+			D-81	-				B-128			++	
C-13			++		D-82	-				B-129	-			
C-14			++		D-83			++		B-130		+		
C-15	-				D-84		+			D-131			++	
C-16		+			D-85	-				D-132			++	
C-17			++		I-86			++		C-133	-			
C-18			++		I-87		+			C-134		+		
C-19			++		I-88	-				C-135	-			
H-41	-				I-89	-				A-136		+		
H-42		+			I-90			++		A-137			++	
H-43	-				I-91		+			A-138	-			
H-44			++		I-92			++		A-139	-			
H-45			++		I-93			++		B-140			++	
I-46	-				G-92				+++	B-141	-			
I-47	-				G-95	-				B-142			++	
I-51			++		G-96		+			B-143	-			
A-52			++		G-97			++		D-144			++	
A-53	-				G-98			++		D-145	-			
A-54		+			A-99	-				D-146			++	
A-55	-				A-100			++		D-147			++	
H-56	-				A-101		+			D-148		+		
H-57				+++	A-102	-				E-149			++	
D-58	-				C-103	-				E-150			++	
D-59			++		C-104	-				E-151	-			
D-60			++		C-105			++		E-152	-			
D-61			++		C-106	-							

**Identification of antagonistic bacteria**

The genomic DNA of the bacterial isolates with antifungal activity were successfully amplified by PCR using primers *27F* and *1492R*. After the electrophoresis of the PCR product on the agarose gel 1%, a fragment of 1500 bp was observed. On the basis of 16s ribosomal RNA sequencing results, they were identified as *Sphingopyxis* sp. LC082 (G-92), *Streptomyces angustmyeticus* (H-57), *Streptomyces libani* (I-65), and *Bacillus subtilis* (I-71). 

**Optimization production of antifungal compounds**

**Figure 2:**
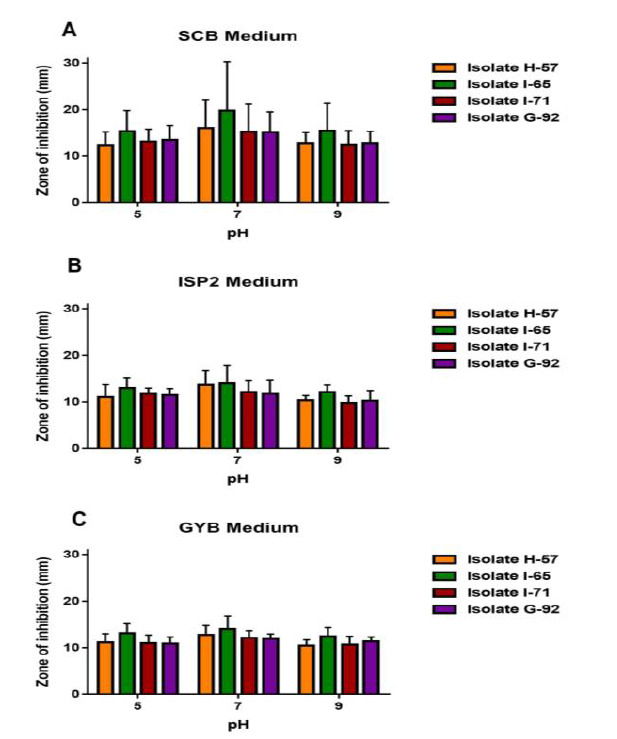
Effect of pH on bioactive metabolite production by four bacterial isolates in SCB, ISP-2B, and GYB media. Data expressed as mean±SD (n=3) *P*˂0.05

**Figure 3:**
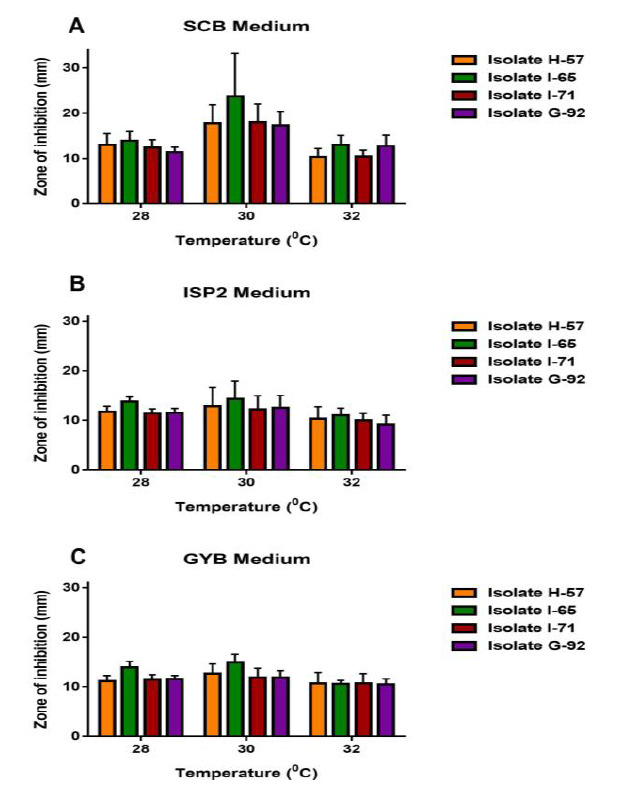
Effect of incubation temperature on bioactive metabolite production by four bacterial isolates in SCB, ISP-2B, and GYB media. Data expressed as mean±SD (n=3) *P*˂0.05

**Figure 4:**
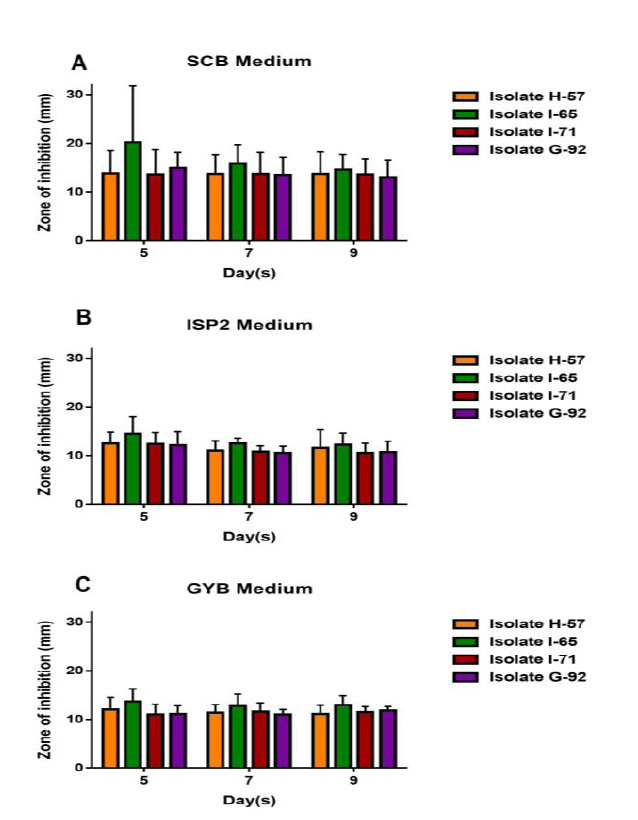
Effect of incubation time on bioactive metabolite production by four bacterial isolates in SCB, ISP-2B, and GYB media. Data expressed as mean±SD (n=3) *P*˂ 0.05

The results obtained from the optimization of production conditions of the antifungal compound based on the type of culture medium, pH, temperature, and incubation time showed that the maximum production of antifungal compounds was in the SCB medium with pH 7 at 30º C for 5 days (Figures 2, 3, and 4). The highest zone of inhibition against *A. fumigatus* (46 mm) was related to the culture filtrate of *S. libani* I-65. 

**Determination of the antimicrobial spectrum of solvent extracts**

**Figure 5:**
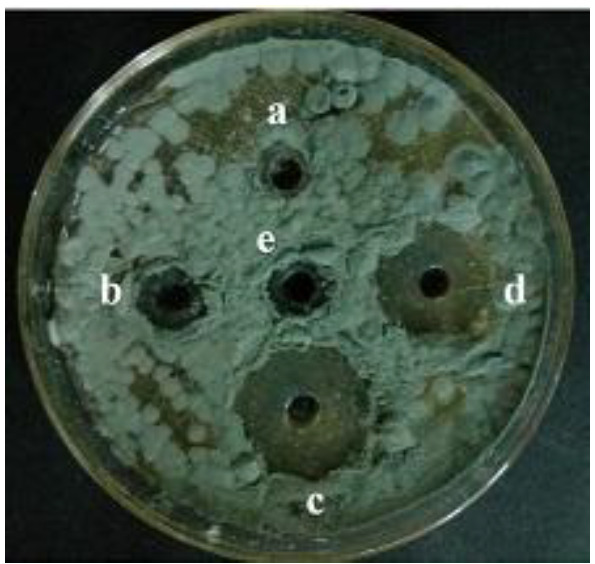
Antifungal activity of solvent extracts from **S. libani** culture against *A. fumigatus* a: AC extract (14 mm); b: MeOH extract (23 mm); c: DCM extract (38 mm); d: EtOAc extract (36 mm); and e: EtOH extract (20 mm)

The antifungal activity of *S. libani* solvent extracts was tested by the agar well diffusion method. All the extracts exhibited varying degrees of inhibition zone against *Aspergillus* species (within the range of 6-38 mm). The DCM extract showed the strongest antifungal activity which had a statistically significant difference with all the other solvent extracts (*P*<0.05). The MeOH and EtOH extracts showed the same level of inhibition against fungal growth (*P*>0.05) and the AC extract showed minimum antifungal activity (*P*<0.05). The DCM, EtOAc, MeOH, and EtOH extracts of *S. libani* SCB culture filtrate inhibited the mycelial growth and sporulation of *A. fumigatus* and *A. niger* (Figure 5). The DCM extract did not affect the growth of *A. flavus* mycelia but inhibited its sporulation. None of the solvent extracts had an inhibitory effect on the growth of examined bacteria (*E. coli* and *S. aureus*) and yeasts (*C. glabrata*, *C. albicans*, and C. neoformans). 

## Discussion

Aspergillosis is an opportunistic fungal infection that has a high mortality rate among fungal diseases in the world. Increase of triazole resistance in clinical isolates of *A. fumigatus* and their limited effect and toxicity are the cause of mortality and morbidity in aspergillosis [ 24 , 25 ]. In these cases, the mortality rate was reported to be 88% [ 24 , 25 ]. Discovery of new medications with more efficiency and less toxicity and resistance is necessary for the treatment of mycotic diseases, especially aspergillosis. 

The soil-inhabiting bacteria, particularly *Strepto-myces* genus, are able to produce diverse bioactive compounds and are used for producing metabolites, such as peptides, macrolides, aminoglycosides, polyenes, polyethers, tetracyclines, and βˍlactams with antimicrobial activity [ 16 , 26 , 27 ]. In several studies, the production of antifungal metabolites, such as bafilomycin A1, oligomycin, geldanamycin, nikkomycin, and reveromycin A and B, has been reported by various strains to belong to the genus of *Streptomyces* [ 27 - 29 ]. Most of these compounds have polyketide and peptide structures and have inhibitory activity against filamentous fungi. Therefore, this study aimed to isolate and identify the antifungal-producing bacteria with emphasis on soil *Streptomyces* since many *Streptomyces* populations inhabit soils. 

In the present study, 110 bacterial isolates were isolated from 45 soils from the northern forests of Iran More than 59.09% of the bacterial isolates inhibited *A. fumigatus* growth, while 40.9% of them did not inhibit fungal growth. Ganesan et al. screened 106 actinomycetes from the forest soil of the Eastern Ghats in India and found that 41.5% of strains exhibited good antimicrobial activity against *Aspergillus brasiliensis* and *B. subtilis* [ 20 ]. Moreover, Khebizi et al. isolated *Streptomyces gancidicus* NBRC 15412 from the Saharan soil sample collected from Algeria and reported that it has significant activity against *Ascobolus carbonarius* M333, *A. niger* OT304, *Fusarium equiseti,* and *Fusarium moniliforme* [ 26 ]. Aliero et al. investigated the antifungal activity in 56 actinomycetes isolated from 22 soil samples and found that two isolates, namely KBRWDSa (FR) and KBMWDSb, inhibited the growth of *C. albicans, Aspergillus* sp., *Fusarium* sp., *Rhizopus* sp. and *Penicillium* sp. [ 30 ]. 

Preliminary screening of antifungal-producing bacteria revealed that four isolates (3.63%) had high inhibitory activity against *A. fumigatus*. The antagonistic bacteria were identified as *Streptomyces libani, Streptomyces angustmyceticus, Bacillus subtilis,* and *Sphingopyxis* sp. As these bacteria have been reported to produce antifungal metabolites, various culture conditions were tested to optimize the production of antifungal metabolites by actinomycetes [ 28 , 30 , 31 , 32 ]. 

In this study, the most antifungal activity was observed in SCB culture medium with pH 7, at 30º C for five days. It has been reported by several researchers that starch and casein are the best sources of carbon and nitrogen for the production of antibiotics [ 20 , 33 ]. Reddy et al. observed that peptone followed by casein can be the best nitrogen source for the production of the antimicrobial metabolite from *Streptomyces rochei* [ 32 ]. In addition, Aliero et al. showed yeast extract starch broth was a good medium for the production of antimicrobial metabolites from an actinomycete isolate KBMWDSb6 [ 30 ]. 

Temperature and pH are important factors for the production of the antifungal metabolite by species of *Streptomyces* genus [ 20 , 30 ]. Reddy et al. found that maximum antifungal metabolite was produced against *C. albicans* at a pH of 7.5 at 32°C for five days [ 32 ]. Kumar et al. reported that *S*. VITSVK5 sp. culture filtrate exhibited the maximum yield of the antifungal compound against *A. fumigatus* in soluble starch broth medium at pH 8.2, 32° C for 72 h [ 33 ]. 

Numerous studies have been conducted on the isolation of antifungal metabolites from various species of *Streptomyces* genus; nevertheless, only few studies have investigated *S. libani*. For instance, Kim et al. reported that *S. libani* has the ability to produce oligomycin in soluble starch broth medium (SSB), at pH 7, 30º C for 12 days. This compound had an inhibitory effect on the mycelia growth of *Cladosporium cucumerinum, Colletotrichum lagenarium,* and *Botrytis cinerea* from 3-5 µg/ml of minimum inhibitory concentration [ 26 ]. 

Antifungal compounds have been extracted from *Streptomyces* culture filtrate by organic solvent. In this study, the DCM extract of the *S. libani* SCB culture showed significant antifungal activity against *Aspergillus* species. Moreover, the maximum zone of inhibition was observed at 38 mm. The EtOAc extract shows less activity than the DCM extract, and EtOH and MeOH extracts showed minimum antifungal activity. However, Bssn et al. reported that EtOAc extract of *Streptomyces lavendulocolor* VHB-9 YMD has significant antifungal activity against *A. niger*, *A. flavus*, Fusarium solani, *P. Citrinum*, and *C. albicans*. The bioactive compounds produced by *S. lavendulocolor* VHB-9 were Bis (7-methyloctyl) phthalate and (Z)-3-aminoacrylic acid [ 34 ]. Aouiche et al. found that *Streptomyces* spp. PAL114 ISP-2 DCM extract has strong antifungal activity against *A. carbonarius*, *A. flavus*, *Fusarium culmorum, Penicillium glabrum,* and *Candida* sp. The inhibitory compounds were identified as saquayamycins A and C [ 35 ]. Khebizi et al. reported that DCM solvent of the *S. gancidicus* beef extract glucose broth (BEG) culture has good antifungal activity against *Fusarium* and *Penicillium* species and two active metabolites were identified as oligomycins E and A [ 26 ]. 

## Conclusion

Bioactive metabolites of microbial origin are now widely considered as novel therapeutics in the treatment of fungal infections. The *S. libani* that was isolated from forest soils of Northern regions of Iran was able to produce antifungal compounds against *Aspergillus* species, particularly the pathogenic species *A. fumigatus*. It is recommended to purify and identify the bioactive antifungal compounds from *S. libani*, which was introduced in this study, as potential sources for designing novel antifungal therapeutics. 

## Authors’ contribution 

M. S. G. managed the project and wrote the first draft of the manuscript. M. R. A. collected the clinical sample, and M. A. performed the experiments. All authors approved the final version of the manuscript. 

## Financial disclosure 

No financial interests have been declared about the material of this manuscript.
